# Laser Ablation-Generated Crystalline Selenium Nanoparticles Prevent Damage of DNA and Proteins Induced by Reactive Oxygen Species and Protect Mice against Injuries Caused by Radiation-Induced Oxidative Stress

**DOI:** 10.3390/ma16145164

**Published:** 2023-07-22

**Authors:** Sergey V. Gudkov, Meng Gao, Alexander V. Simakin, Alexey S. Baryshev, Roman V. Pobedonostsev, Ilya V. Baimler, Maksim B. Rebezov, Ruslan M. Sarimov, Maxim E. Astashev, Anastasia O. Dikovskaya, Elena A. Molkova, Valery A. Kozlov, Nikolay F. Bunkin, Mikhail A. Sevostyanov, Alexey G. Kolmakov, Mikhail A. Kaplan, Mars G. Sharapov, Vladimir E. Ivanov, Vadim I. Bruskov, Valery P. Kalinichenko, Kuder O. Aiyyzhy, Valery V. Voronov, Nuttaporn Pimpha, Ruibin Li, Georgy A. Shafeev

**Affiliations:** 1 Prokhorov General Physics Institute of the Russian Academy of Sciences, 38 Vavilova St., 119991 Moscow, Russia; avsimakin@gmail.com (A.V.S.); aleksej.baryshev@gmail.com (A.S.B.); pobedonoscevroman@rambler.ru (R.V.P.); ilyabaymler@yandex.ru (I.V.B.); rebezov@yandex.ru (M.B.R.); rusa@kapella.gpi.ru (R.M.S.); astashev@yandex.ru (M.E.A.); dikovskayaao@gmail.com (A.O.D.); bronkos627@gmail.com (E.A.M.); v.kozlov@hotmail.com (V.A.K.); nbunkin@mail.ru (N.F.B.); iwe88@rambler.ru (V.E.I.); kuder_1996@mail.ru (K.O.A.); voronov@lst.gpi.ru (V.V.V.); shafeev@kapella.gpi.ru (G.A.S.); 2 Russian Scientific-Research Institute of Phytopathology of Russian Academy of Sciences, 143050 Big Vyazemy, Russia; cmakp@mail.ru (M.A.S.); kalinitch@mail.ru (V.P.K.); 3 Institute of Biology and Biomedicine, Lobachevsky State University of Nizhny Novgorod, 603022 Nizhny Novgorod, Russia; 4 State Key Laboratory of Radiation Medicine and Protection, School for Radiological and Interdisciplinary Sciences (RAD-X), Collaborative Innovation Center of Radiation Medicine of Jiangsu Higher Education Institutions, Suzhou Medical College, Soochow University, Suzhou 215123, China; gaomy@iccas.ac.cn (M.G.); liruibin@suda.edu.cn (R.L.); 5 Institute of Cell Biophysics of the Russian Academy of Sciences, Federal Research Center “Push-chino Scientific Center for Biological Research of the Russian Academy of Sciences”, Institutskaya St., 3, 142290 Pushchino, Russia; sharapov.mars@gmail.com; 6 Department of Fundamental Sciences, Bauman Moscow State Technical University, 2-nd Baumanskaya Str. 5, 105005 Moscow, Russia; 7 A. A. Baikov Institute of Metallurgy and Materials Science (IMET RAS) of the Russian Academy of Sciences, Leninsky Prospect, 49, 119334 Moscow, Russia; kolmakov@imet.ac.ru (A.G.K.); 79031927386@yandex.ru (M.A.K.); 8 Institute of Theoretical and Experimental Biophysics of the Russian Academy of Sciences, Institutskaya St. 3, 142290 Pushchino, Russia; bruskov_vi@rambler.ru; 9 Institute of Fertility of Soils of South Russia, 346493 Persianovka, Russia; 10 National Nanotechnology Center (NANOTEC), National Science and Technology Development Agency (NSTDA) 111, Phahonyotin Rd, Klong Luang 12120, Thailand; nuttaporn@nanotec.or.th

**Keywords:** radioprotection, radiation injuries, oxidative stress, laser ablation, crystalline selenium nanoparticles, DNA and protein damage

## Abstract

With the help of laser ablation, a technology for obtaining nanosized crystalline selenium particles (SeNPs) has been created. The SeNPs do not exhibit significant toxic properties, in contrast to molecular selenium compounds. The administration of SeNPs can significantly increase the viabilities of SH-SY5Y and PCMF cells after radiation exposure. The introduction of such nanoparticles into the animal body protects proteins and DNA from radiation-induced damage. The number of chromosomal breaks and oxidized proteins decreases in irradiated mice treated with SeNPs. Using hematological tests, it was found that a decrease in radiation-induced leukopenia and thrombocytopenia is observed when selenium nanoparticles are injected into mice before exposure to ionizing radiation. The administration of SeNPs to animals 5 h before radiation exposure in sublethal and lethal doses significantly increases their survival rate. The modification dose factor for animal survival was 1.2. It has been shown that the introduction of selenium nanoparticles significantly normalizes gene expression in the cells of the red bone marrow of mice after exposure to ionizing radiation. Thus, it has been demonstrated that SeNPs are a new gene-protective and radioprotective agent that can significantly reduce the harmful effects of ionizing radiation.

## 1. Introduction

The search for effective radioprotective substances for use in various scenarios of the interaction of ionizing radiation with the body has been going on for more than seventy years [1]. Currently, various classes of chemical compounds are known that can protect biological objects from the short-term and long-term effects of ionizing radiation when they enter the body, both before and after irradiation [2]. For most radioprotective substances, the dose change factor, time of administration, tissue specificity, toxicity, mechanisms of action, and areas of practical application are known [3]. The following classes of radioprotective drugs are usually distinguished [4]: sulfhydryl compounds, antioxidants, angiotensin-I-converting enzyme inhibitors, modulators and cytokines, prostaglandins, metal salts and metallothionein, DNA binding agents, compounds that cause hypoxia, RNA, RNA hydrolysates, nucleosides, fullerenes, adsorbents, and selenium compounds. Selenium is one of the vital elements for all living beings, as it plays an important role in the functioning of the evolutionarily ancient antioxidant enzymes known as glutathione peroxidases [5]. The activity of glutathione peroxidases correlates with the body’s resistance to oxidative stress. This property of selenium is used in the prevention of a number of diseases associated with the development of oxidative stress (cardiovascular diseases, arthritis, muscular dystrophy, cystic fibrosis, etc.) in clinic [6]. For example, selenium stimulates the immunity of mammals [7] and has anticancer properties [8], including the ability to overcome the multidrug resistance of tumors [9]. These properties of selenium are probably associated with selenium-containing antioxidant proteins of the endoplasmic reticulum [10]. In addition, selenium compounds, due to direct interaction, effectively reduce the degree of toxicity of a number of heavy metals and toxins [11].

Oxidative stress is a pathological state of the body, which is characterized by a change in redox homeostasis and the prevalence of oxidative and destructive processes [12,13,14]. Ionizing radiation is a classical influence leading to the beginning of the formation of oxidative stress. As a result, one kind of the most effective radioprotectors (chemical compounds that protect against radiation) is antioxidants, which also include selenium compounds [15,16]. The radioprotective effect of selenium-containing compounds was first discovered in 1964 [17]. Later, it was demonstrated that selenium compounds not only provide protection against radiation on their own but also have the ability to modify the protective effects of other radioprotectors [18,19]. In practice, inorganic selenium compounds (mainly selenites and selenates), selenium-containing amino acids (selenocysteine, selenometeonine), and selenium-containing proteins are currently used. Such methods of selenium delivery to the body are usually characterized by a low rate of absorption in the gastrointestinal tract and also have significant toxicity [20]. In this regard, it is quite important to create compositions and systems that make selenium more bioavailable and promote its controlled and gradual release in the body. Recently, selenium-containing nanoparticles have been claimed to have various biological activities in metabolic regulation [21]. In this work, a technology for obtaining selenium nanoparticles (SeNPs) with controlled sizes, shapes, properties, and significant radioprotective properties has been created.

## 2. Materials and Methods

### 2.1. Preparation and Characterization of Selenium Nanoparticles

Selenium nanoparticles were obtained using the method of laser ablation in liquid. A massive selenium target was attached to the bottom of the experimental cell. Liquid was poured into the cuvette so that there was a layer of liquid several millimeters high above the massive target. In this process, deionized water was used as a liquid. The method of laser ablation in a liquid makes it possible to create nanoparticles with controlled characteristics. An ytterbium fiber laser with variable pulse duration (λ = 1064 nm, τ = 4–200 ns, f = 20 kHz, P = 20 W, Ep = 1 MJ) was used for selenium ablation. Using the heads of the Ateko-TM 2D galvanic scanner (Ateko, Moscow, Russia), the laser beam moved along the surface of a massive selenium target [22].

**Figure 1:**
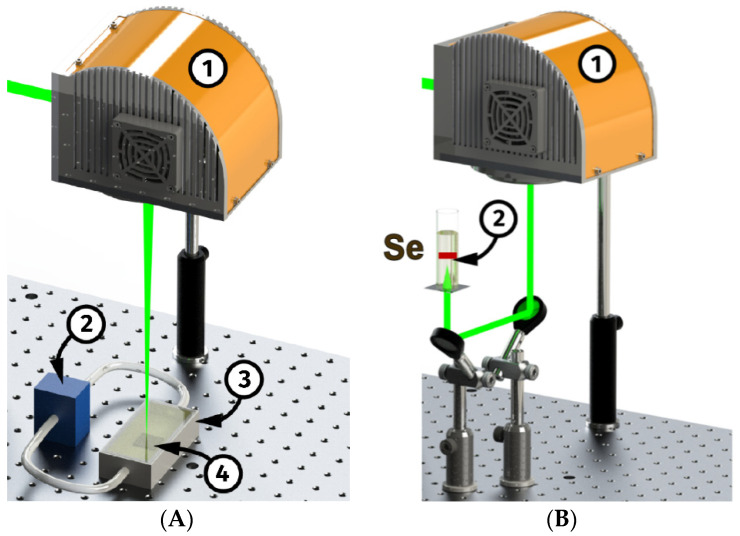
Scheme of production of selenium nanoparticles using laser ablation. (**A**) Production of selenium particles in a flow cuvette (scanner head (1), circulation pump (2), continuous-flow cell (3), bulk Selenium target (4)). (**B**) Production of selenium nanoparticles using laser fragmentation (scanner head (1), cell with suspension of nanoparticles (2)).

Laser ablation of a solid selenium target in a flow-through cell (Figure 1A): this approach makes it possible to obtain large-volume colloidal solutions of nanoparticles, which can thereafter be partially evaporated in order to increase the nanoparticle concentration. The advantage of using high energy per pulse fiber laser allows the generation of a high concentration of Se nanoparticles with wide size distribution, including micro- and nanoparticles. The preparation of such particles was subjected to further laser fragmentation [23]. In this case, suspensions of selenium micro-powders were irradiated by a laser beam entering the cells through a glass window from below (Figure 1B). This version of the setup made it possible to control the nanoparticle size distribution by varying the focusing depth in the solution and the fragmentation time. The diameters of SeNPs were analyzed using a disc centrifuge DC 24000 (CPS Instruments, Prairieville, LA, USA) according to the protocol described earlier [24]. The data on the size of nanoparticles obtained using a disk centrifuge were confirmed by the method of dynamic light scattering, using a DLS Malvern Zetasizer Ultra Red Label 10 (Malvern, UK) with multi-angle dynamic light-scattering technology (Malvern, PA, USA). The DLS data were obtained at several scattering angles; the data were calculated according to the algorithm proposed earlier [25]. The morphology of SeNPs was inspected using a 200FE TEM ((Carl Zeiss Microscopy GmbH, Jena, Germany). Samples for microscopy were prepared on gold microscopic grids [26]. The crystalline structure of selenium nanoparticles was studied using a Bruker AXS P4 X-ray diffractometer (Bruker, Billerica, MA, USA). The spectral characteristics of selenium nanoparticles were studied using a USB 3000T spectrometer (Ocean Optics, Orlando, FL, USA).

### 2.2. Animals

In the experiments, male white Kv:SHK mice were used at the age of 5–6 weeks and weighing from 21 to 24 g (nursery Kryukovo, Russian Academy of Medical Sciences). The animals were kept in a vivarium and fed a standard diet with free access to granulated commercial mouse food and tap water. The work with laboratory animals was carried out in accordance with the provisions of the European Convention for the Protection of Vertebrate Animals used for Experiment and other Scientific Purposes (1986) and the ICB RAS guidelines for working with laboratory animals No. 57.30.12.2011.

### 2.3. The Effect of Ionizing Radiation

A therapeutic X-ray machine RUM-15 (Mosrentgen, Moscow, Russia) was used for irradiation with ionizing radiation. The main characteristics of the X-ray machine were as follows: dose rate of 1 G/min, focal length 37.5 cm, current 20 mA, voltage 200 kV. Exposure to ionizing radiation at a dose of 15 Gy was applied to cell cultures. Exposure to ionizing radiation at a dose of 1.5 to 8 Gy was applied to mice.

### 2.4. Survival of Mice

Selenium nanoparticles were dissolved at 37 °C in isotonic glucose solution immediately before the experiment. Before irradiation, the mice were injected intraperitoneally (IV) with a colloidal solution of selenium nanoparticles in a volume of 0.2 mL. The control group received an isotonic solution. After that, the mice were kept in polypropylene cages with free access to water and food before exposure to ionizing radiation. The number of surviving animals was studied for 30 days after irradiation with a frequency of 24 h [27].

### 2.5. Survival of Cell Cultures

Human neuroblastoma cell culture (SH-SY5Y) and primary mouse fibroblast culture (PCMF) were used in the experiments. The SeNPs were added into culture medium under sterile conditions in the case of experiments with 24 h pre-incubation with SeNPs. Then, the cell cultures were washed after the pre-incubation with Hank’s balanced salt solution and used in experiments. Details of the work with cell cultures are described earlier [28].

### 2.6. Hematology

Peripheral blood was taken from the tail vein of mice. No fewer than 100 leukocytes were scored on each smear to determine granulocyte count [29]. The number of platelets per 1000 erythrocytes was counted on the same smear, and then normalized to a liter of blood. All experimental procedures were described in detail previously [30].

### 2.7. Micronucleus Test

SeNPs in isotonic solution were administered to mice 5 h before exposure to ionizing radiation. Samples were taken 28 h after irradiation. At this time, the highest level of polychromatic erythrocytes (PE) with micronuclei (MYA) was observed in the red bone marrow. Bone marrow cell preparations were made according to the method developed by us earlier [31]. The procedures related to the preparation and staining of histological preparations were described earlier [32].

### 2.8. Measurement of Protein Oxidation Levels

The concentration of carbonyl groups was evaluated using the calorimetric method. The method is based on a color reaction in which the carbonyl groups of proteins interact with a dye, 2,4-dinitrophenylhydrazine (DNFG). Mass screening was carried out on 96-well round-bottom plates, as described earlier [20].

### 2.9. Real-Time PCR

The primers synthesized earlier in the company “Eurogen” (Moscow, Russia) and the procedures used in the analysis are described earlier in the publication [33]. Deionized water was used as a negative control.

### 2.10. Measurement of the Concentration of Hydrogen Peroxide and Hydroxyl Radicals

The concentration of hydroxyl radicals was determined using the reaction with coumarin-3-carboxylic acid (3-CCA) (Aldrich, Burlington, MA, USA), the hydroxylation product of which, 7-hydroxycoumarin-3-carboxylic acid, is a convenient fluorescent probe for determining the formation of these radicals [34]. The conditions for the experiment were described in detail earlier [35]. Hydrogen peroxide concentration was determined using the method of enhanced chemiluminescence in the luminol-p-iodophenol-peroxidase system, as published earlier [36]. The sensitivity of the methods is approximately 0.1 nM [37,38].

## 3. Results

**Figure 2:**
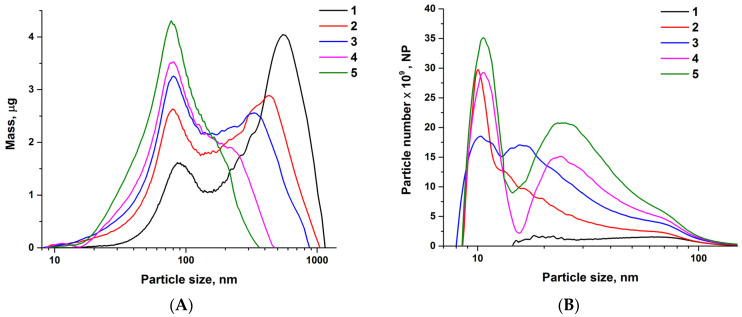

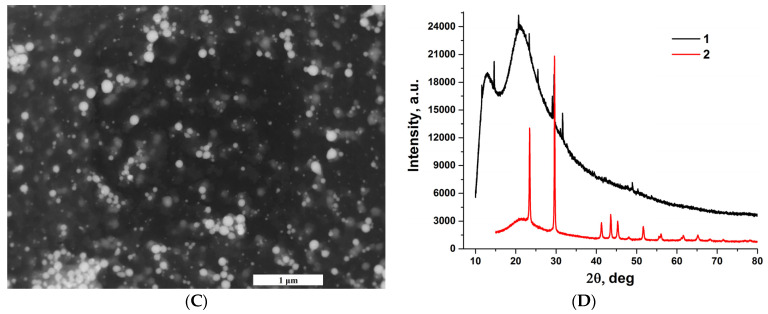
Physical and chemical characteristics of SeNPs. (**A**,**B**) Mass and number distribution of SeNPs on their size at various laser fragmentation times. Initial suspension by laser ablation of Se target in the flow cell (1), fragmentation time 30 min (2), 90 min (3), 150 min (4), and 210 min (5). (**C**) TEM view of Se nanoparticles. General view, scale bar denotes 1 μm. (**D**) X-ray diffraction patterns of Se nanoparticles. 1—particles obtained directly after laser ablation in flow cuvette. 2—particles obtained after laser fragmentation. No other Se-containing compounds, such as Se oxide, were detected within the sensitivity of X-ray analysis.

The change in the weight and number distributions of selenium nanoparticles by size depends on the time of irradiation of a colloidal solution of selenium nanoparticles and shows that before irradiation, the bulk of the particles is concentrated in particles with a diameter of 500 nm and more (Figure 2A,B). As irradiation is observed, smaller and smaller nanoparticles begin to predominate in the colloid. With sufficiently long irradiation, most of the mass of the colloid falls on nanoparticles with a diameter of approximately 80 nm. At the same time, the largest number of nanoparticles have a size of approximately 10 nm. The images obtained by TEM show SeNPs with sizes of approximately 80–100 nm and 10–15 nm. Particle sizes that can be calculated from TEM micrographs (Figure 2C) match the diameter distribution plotted by the CPS analytical centrifuge. One fraction of SeNPs is easily separated from another fraction of nanoparticles by centrifugation. A fraction of nanoparticles with a small size was used in the experiments. Figure 2D shows X-ray diffraction patterns of two samples of selenium nanoparticles. Peaks at 23.4 and 29.6 deg are observed on the diffraction patterns of selenium nanoparticles, corresponding to two phases of selenium: hexagonal (no. 3-363 according to the PCPDFWIN database) and monoclinic (no. 24-1202 according to the PCPDFWIN database). The preparation of selenium nanoparticles is homogeneous and highly stable. No precipitation of SeNPs was observed for several days at room temperature. The zeta potential of the nanoparticles is −30 mV.

The effect of selenium nanoparticles on the generation of hydroxyl radicals in aqueous colloids under the action of ionizing radiation has been studied. It has been shown that hydroxyl radicals of the order of 240 nM/Gy are formed in water under the action of ionizing radiation. At a concentration of 100 mg/L in SeNPs in an aqueous colloid, approximately 180 nM/Gy is formed under the action of ionizing radiation, and at a concentration of 200 mg/L in SeNPs, approximately 150 nM/Gy. The influence of selenium nanoparticles on the generation of hydrogen peroxide in aqueous colloids under the action of ionizing radiation was also studied. It has been shown that approximately 80 nM/Gy of hydrogen peroxide is formed in water under the action of ionizing radiation. At a concentration of 100 mg/L in SeNPs in an aqueous colloid, 61 nM/Gy is formed under the action of ionizing radiation, and at a concentration of 200 mg/L in SeNPs, approximately 52 nM/Gy.

Toxicological tests of the obtained nanoparticles were carried out. It was shown that SeNPs do not exhibit any toxic effects upon a single exposure to SH-SY5Y at a concentration of up to 125 mg/kg or on a PCMF at a concentration of up to 75 mg/kg. At a SeNPs concentration of 350 mg/kg, less than 30% of non-viable cells are observed in the SH-SY5Y cell culture, and approximately 70% of non-viable cells are observed in the PCMF culture with the same exposure. Thus, the SeNPs obtained by us do not exhibit significant toxic properties, in contrast to molecular selenium compounds.

**Figure 3:**
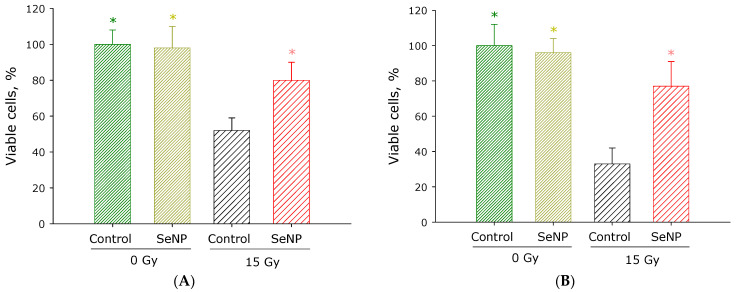
Effect of SeNPs on the survival of cell cultures after exposure to ionizing radiation at a dose of 15 Gy. (**A**) Culture of human neuroblastoma cells (SH-SY5Y). (**B**) Primary culture of mouse fibroblasts (PCMF). Asterisks indicate a significant difference at 5% level (Student’s unpaired *t*-test) in comparison with the irradiation control. *p* < 0.01.

Can the SeNPs we have obtained neutralize the negative effects of oxidative stress? To answer this question, the effects of SeNPs on the survival of SH-SY5Y and PCMF cells after exposure to ionizing radiation at a dose of 15 Gy were investigated (Figure 3). It was shown that the administration of SeNPs can significantly increase the viabilities of SH-SY5Y and PCMF cells after radiation exposure from 50% to 80% and 30% to 80%, respectively. Thus, SeNPs protect cell cultures from the destructive effect of ionizing radiation, but studies on cell cultures can only be considered as primary screening.

**Figure 4:**
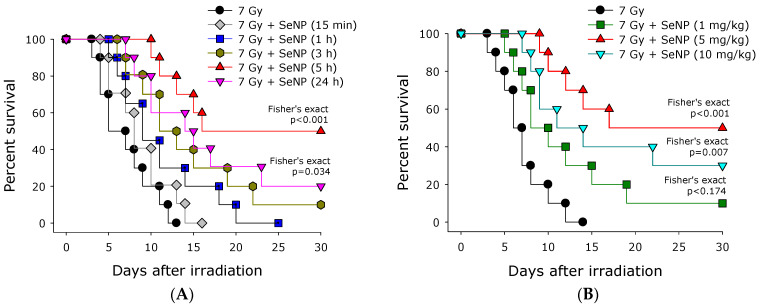
Effect of SeNPs on the survival of animals after irradiation. (**A**) Effect of SeNPs (5 mg/kg) upon intravenous injection at various times before 7 Gy irradiation on the survival of animals. (**B**) Effect of SeNPs upon intravenous injection at various concentrations 5 h before 7 Gy irradiation on the survival of animals.

The survival rate of mice treated with SeNPs before exposure to ionizing radiation at a lethal dose of 7 Gy was studied (Figure 4A). It was found that the average survival of mice not receiving SeNPs is 6 days, and the maximum survival of mice not receiving SeNPs is 13 days. When SeNPs was administered 15 min before exposure to ionizing radiation, mice had an average survival of 8 days, while the maximum survival of animals increased to 16 days. When SeNPs were administered 1 h before exposure to ionizing radiation, mice had an average survival rate of 10 days, while the maximum survival rate of animals increased to 25 days. When SeNPs were administered 3 h before exposure to ionizing radiation, mice lived up to 30 days. When administered 5 h before, approximately 50% of animals remained alive, while when administered 1 day before exposure to radiation, approximately 20% of animals remained alive. With the introduction of SeNPs 36 and 48 h before irradiation, 20 and 15% of animals survived until the end of the experiment.

The survival rate of mice after the administration of SeNPs 5 h before X-ray irradiation at a lethal dose of 7 Gy in various concentrations was studied (Figure 4B). A slight radioprotective effect was observed when SeNPs were administered at a concentration of 1 mg/kg. At the same time, only 10% of mice remained alive by 30 days after irradiation. It was found that by the 30th day approximately half of the mice remained alive after irradiation at a dose of 7 Gy with the introduction of selenium nanoparticles at a concentration of 5 mg/kg. The magnitude of the radioprotective effect decreased slightly with an increase in the concentration of SeNPs to 10 mg/kg, while by 30 days approximately 30% of the animals remained alive.

It is known that after exposure to ionizing radiation in experimental animals, body weight changes, and animals begin to consume less water and food. In this regard, the consumption of water and food, as well as the change in the mass of the mice, was investigated. During the experiment, the consumption of food and water by intact mice that received and did not receive nanoparticles did not change significantly (Table 1). When mice were exposed to ionizing radiation at a dose of 7 Gy, there was a decrease in consumption of approximately 30–40% for food and 45–60% for water. At the same time, in irradiated mice receiving SeNPs before irradiation, the consumption of food and water was on average higher compared to irradiated mice not receiving selenium nanoparticles. The irradiated mice that received nanoparticles had their food and water intake normalized by the 15th day. In separate experiments, we investigated the acute toxicity of SeNPs at a concentration of 10 mg/kg. It has been established that even at such concentrations there are no changes in the skin or in water and food consumption.

**Figure 5:**
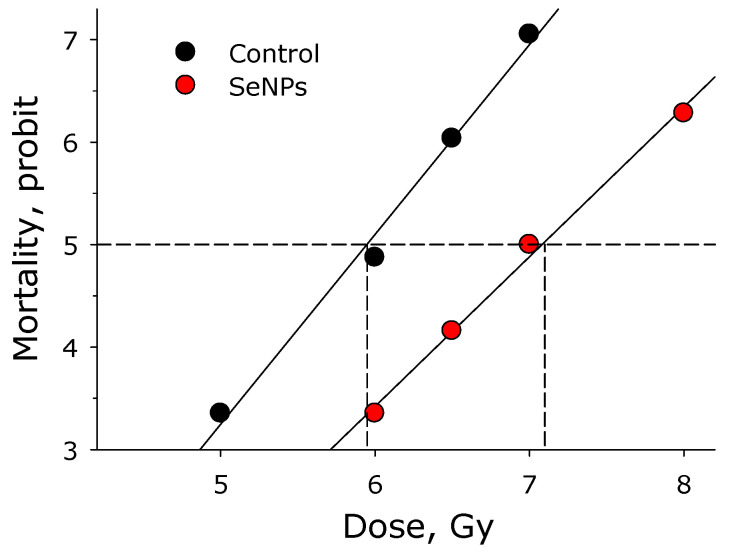
Effect of intravenous administration of SeNPs (5 mg/kg) 5 h before irradiation on the survival of mice. The graph is presented as a dose-mortality dependency. The x-axis—logarithmic function; the y-axis—probit function. Each point on the graph represents the data from at least 20 animals.

In order to numerically characterize the radioprotective effect of selenium nanoparticles, the dose reduction factor (DRF) was calculated (Figure 5). For this, LD_50/30_ values were calculated for mice with selenium nanoparticles injected at a concentration of 5 mg/kg 5 h before exposure to ionizing radiation and mice irradiated and not exposed to nanoparticles. It was found that LD_50/30_ for mice that did not receive selenium nanoparticles was 5.9 Gy. LD_50/30_ for mice treated with SeNPs was 7.1 Gy. Thus, the DRF for selenium nanoparticles is approximately 1.2.

**Figure 6:**
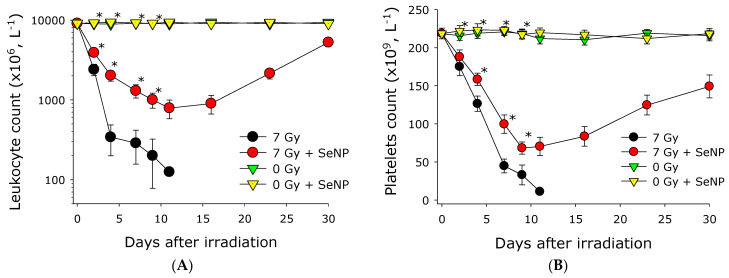
Effect of SeNPs (5 mg/kg) administered intraperitoneally 5 h before X-ray irradiation (7 Gy) on leukocyte (**A**) and platelet (**B**) content in peripheral blood of exposed mice in post-irradiation period. Data points represent median with lower quartile and upper quartile for 2–5 animals. Statistically significant differences between irradiation control group and the other groups (Mann–Whitney U test, *p* < 0.05) are marked by asterisks.

When small rodents are exposed to ionizing radiation in the dose range from 3 to 10 Gy, hematopoietic syndrome is observed. It is the hematopoietic syndrome that is often the cause of death in such conditions. In this regard, the dynamics of changes in the content of leukocytes and platelets in the peripheral blood of mice receiving selenium nanoparticles and mice not receiving selenium nanoparticles and exposed to ionizing radiation at a dose of 7 Gy, was studied. The leukocytes and platelets numbers in the group of unirradiated animals practically did not change throughout the experiment. In the groups of animals that were not administered SeNPs and those that received SeNPs prior to irradiation, there was a reduction of approximately 98% and 85% in the number of leukocytes, respectively, by the 8th day of the experiment. (Figure 6A). Subsequently, in the group of irradiated mice, the number of leukocytes continued to decrease significantly; this happened until the death of the animals. In the group of animals that were injected with SeNPs, by day 16 after irradiation, the number of leukocytes stabilized. At later times, the number of leukocytes began to recover. By the 30th day, there was a significant recovery in the number of leukocytes.

The content of platelets in the peripheral blood of irradiated mice receiving and not receiving selenium nanoparticles is shown in Figure 6B. By the 8th day of the experiment, the number of platelets in the blood of irradiated animals decreased by almost 95% compared to the baseline (before the experiment). At the same time, by the 8th day of the experiment, the number of platelets in the blood of irradiated animals receiving SeNPs decreased by only 70% compared to the initial one. Subsequently, the platelet count in the peripheral blood increased; by the 30th day the level of platelets reached 65% of the control level. Consequently, the findings demonstrate that SeNPs effectively mitigate radiation-induced thrombo- and leukopenia, potentially influencing the animals’ survival following exposure to X-ray radiation.

**Figure 7:**
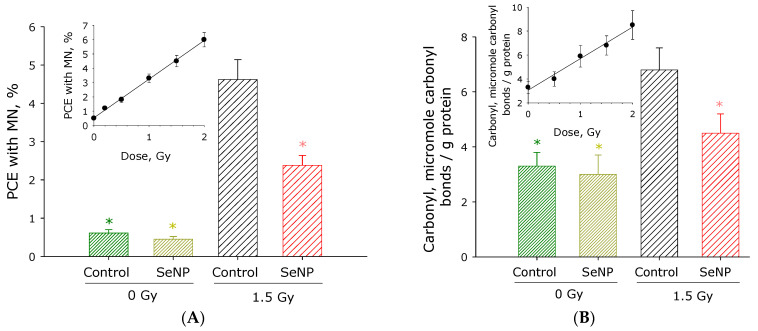
Effect of SeNPs (5 mg/kg) injected i.p. to mice 5 h prior to their irradiation with 1.5 Gy of X-rays on the formation of PCE with MN in the bone marrow cells (**A**) and carbonyl derivatives in blood plasma proteins (**B**) of the animals. The data were obtained by performing a micronucleus test and are given as means ± SEM (n = 5). Statistically significant differences between irradiation control group and the other groups (Mann–Whitney U test, *p* < 0.05) are marked by asterisks. Inset: Dose dependence of the formation of PCE with MN or carbonyl derivatives upon X-ray irradiation.

The effect of SeNPs on damage to blood plasma proteins and the DNA of red bone marrow cells under the exposure of X-ray radiation was studied. It is known that red bone marrow is one of the most radiosensitive tissues. A micronucleus test was used, and the effect of SeNPs (with a single intraperitoneal injection 5 h before irradiation) on the percentage of polychromatophilic erythrocytes (PCE) with micronuclei (MN) in the red bone marrow of irradiated mice with X-rays at a dose of 1.5 Gy was studied (Figure 7A). No significant change in the percentage of PCE with MN was observed when SeNPs (5 mg/kg) were administered to intact animals. After exposure to ionizing radiation on animals, the percentage of PCE with MN increased more than 9 times from 0.5% at 0 Gy to 4.5% at 1.5 Gy. In animals that were injected with SeNPs (5 mg/kg) before irradiation, the percentage of PCE with MN decreased by 50% compared to the control group irradiated with a dose of 1.5 Gy. When SeNPs were administered to animals 5 h before irradiation at a concentration of 1 or 10 mg/kg, the percentage of PCE with MN decreased by only 25 and 45%, respectively. When animals are administered SeNPs (5 mg/kg) 1, 3, 5, 36, or 48 h before irradiation, the percentage of PCE with MN decreases by 10, 25, 40, 35, or 25%, respectively.

The effect of SeNPs (with a single intraperitoneal injection 5 h before irradiation) on damage to blood plasma proteins was studied (Figure 7B). It was shown that the administration of SeNPs (5 mg/kg) to intact animals did not result in significant changes in the level of carbonylation of blood plasma proteins. After exposure to ionizing radiation at a dose of 1.5 Gy on animals, the level of protein carbonylation increased by approximately two times. When SeNPs (5 mg/kg) were administered to animals before irradiation, the level of carbonyls decreased by approximately 60% compared to the control irradiated at a dose of 1.5 Gy. When SeNPs were administered to animals 5 h before irradiation at a concentration of 1 or 10 mg/kg, the percentage of carbonyl levels decreased by only 20 and 50%, respectively. When SeNPs (5 mg/kg) were administered to animals 1, 3, 5, 36, or 48 h before irradiation, the percentage of PCE with MN decreased by 10, 20, 45, 30, or 20%, respectively. Thus, it has been shown that SeNPs are able to protect proteins and DNA from the harmful effects of ionizing radiation.

Thus, SeNPs can significantly increase the survival rate of animals when administered before exposure to ionizing radiation. It can be assumed that such effects are not only related to the physicochemical processes occurring under the action of ionizing radiation, but that they also affect the signal-regulatory systems of cells. To test this assumption, the profile of cell expression was investigated (Table 2). After 24 h of administration with SeNPs, most genes’ expression levels slightly changed (within 25–50%). Additionally, the level of mRNA of the NRF2 gene decreased by 200%, and the level of mRNA of the Prx6 gene increased by almost one order. It can be assumed that the introduction of selenium nanoparticles changes the redox homeostasis inside cells, which leads to a decrease in the level of the main regulator of the expression of genes responsible for the antioxidant response (NRF2). Moreover, a decrease in the level of NRF2 may be related to an increase in NF-κB gene level, the expression of which after the introduction of SeNPs increased by more than 50%. This is supported by an increase in the expression level of the IL-6 gene. The relationship between the expression levels of the NRF2 and NF-κB genes, as well as IL-6, is described in detail in [39].

Significant changes in the expression of most genes were observed one day after exposure to ionizing radiation. Only 4 out of 13 genes did not change the level of transcription. The level of mRNA encoding the NF κB protein increased 10 times, Prx6 12 times, and XRCC4 3 times. For the HO-1, HSP90, NRF2, Catalase, and AP-1 genes, a decrease in the expression level by 6, 2.5, 3, 2, and 4 times, respectively, was observed. In irradiated animals treated with SeNPs, in red bone marrow cells, relative to non-irradiated control, an increase in the mRNA level of NFκB genes by 4 times, SOD2 and Xrcc4 by more than 2 times, Prx6 and Xrcc5 by 3 times was observed. In animals treated with SeNPs and irradiated, compared with irradiated animals not treated with SeNPs, a significant normalization of mRNA levels of the HO-1, HSP90, NRF2, Catalase, TNF-α, AP-1, Ki67, and IL6 genes was observed. While mRNA levels of HO-1, HSP90, NRF2, catalase, TNF-α, AP-1, Ki67, and IL6 genes were normalized in SeNP-treated animals, it is worrying that NFKB gene levels increased after SeNP treatment. This state of affairs can potentially lead to risks, which are considered and discussed in detail in the papers [40,41,42].

## 4. Discussion

Previously, a number of methods for obtaining selenium nanoparticles using laser ablation have been proposed. One of the first works on the preparation of selenium nanoparticles using laser ablation was published relatively recently in 2002 [43]. The work shows the fundamental possibility of obtaining nanoparticles. In later works, different teams tried to obtain smaller and smaller nanoparticles [44,45,46]. It should be noted that the problem of obtaining selenium nanoparticles of the desired shape, surface topology, and composition was solved [47]. The problem of nanoparticle size remained unresolved. Researchers tried to solve the problem of obtaining nanoparticles of the required size by using new ablation schemes and changing the laser parameters, sometimes even by completely replacing the laser itself. In this work, for the first time, a technology has been developed for obtaining selenium nanoparticles with the necessary characteristics using only a combination of two parallel ablation schemes (without any rearrangements and changes). The technology is based on the alternating use of ablation in the flow cell and fragmentation (Figure 1); it is possible to assemble two systems, either in one circuit or separately. The technology allows real-time spectral control of the size of nanoparticles (Figure 2), although we additionally confirmed the sizes using an analytical disk centrifuge and HR-TEM. It should be noted that laser ablation produces crystalline SeNPs devoid of any impurities (Figure 2).

It is known that selenium nanoparticles in the body are, in a slow and controlled way (depending on size), capable of releasing bioavailable selenium. At the same time, the toxicity of selenium nanoparticles is 50–100 times lower compared to the molecular forms of selenium. For example, LD_50_ for the oral intake of molecular forms of selenium is approximately 2–6 mg/kg [48]. For SeNPs, the oral LD_50_ is 140–200 mg/kg [49,50]. In our studies, a SeNPs concentration of the order of 5 mg/kg was used, that is, several tens of times lower than LD_50_. It has been shown that SeNPs (5 mg/kg) protect DNA and cell proteins from the harmful effects of ionizing radiation, reducing the damaging effect by almost half (Figure 7). It is known that the use of nanoparticles proved to be effective in protecting biomacromolecules from the damaging effects of ultraviolet light [51], chromium ions [52], and cisplatin [53]. Additionally, nanomaterials can also provide therapeutic effects on hyperoxide microenvironments [53] and the chemotherapy resistance [54] of cancer.

It is known that molecular forms of selenium, even at low concentrations (2 mg/kg), can lead to the development of leukopenia in animals [55]. We have shown that the administration of SeNPs to animals at a concentration of 5 mg/kg does not significantly affect the presence of leukocytes or platelets in the bloodstream. At the same time, SeNPs significantly reduce the acuteness of radiation-induced leuko/thrombopenia (Figure 6). It should be noted that it was previously reported that molecular forms of selenium reduced the severity of leukopenia under the action of cisplatin by only 40% [56]. In our study, SeNPs reduce the severity of leukopenia by several times (Figure 6).

Selenium compounds effectively protect cells and animals from the harmful effects of X-ray radiation (Figure 3, Figure 4 and Figure 5). Previously, a number of studies have shown that the introduction of selenium compounds 24 h before irradiation is more effective than 1 h before [48]. Our studies also confirmed that 24 h administration is more effective than 1 h administration (Figure 4). More interesting is the fact that SeNPs are effective for almost 48 h after administration. This is one of the most prolonged protective effects known today. It is assumed that several hours are needed for selenium compounds to be metabolized to selenomethionine, an increase in the content of which leads to an increase in the expression of antioxidant enzymes. Perhaps such a prolonged radioprotective effect of selenium compounds is due to the duration of their metabolism (conversion) into selenomethionine, which is a part of the most important antioxidant enzymes—selenium-containing glutathione peroxidases [57,58]. It is known that the injection of selenium nanoparticles into mice leads to a multiple increase in the expression of peroxiredoxins [59]. Peroxiredoxins have a significant radioprotective potential [60], and it is possible that the radioprotective properties are not least associated with changes in the expression levels of these antioxidant proteins.

Thus, a technology for obtaining nanosized crystalline selenium of given sizes has been created. The introduction of such nanoparticles into the animal organism protects proteins and DNA from radiation-induced damage, decreases the seriousness of radiation-induced thrombo- and leukopenia, and also significantly increases the survival rate of animals exposed to sublethal and lethal doses of X-ray radiation. The radioprotective action of SeNPs is prolonged for up to 48 h. It should be noted that nanosized selenium can be used not only for protection against radiation or as a therapeutic agent without significant side effects in medicine, but also in the most unexpected areas. For example, it has recently been shown that selenium deficiency in the body correlates with a sharp increase in mortality from COVID-19. Statistics for China, the inhabitants of some areas of which are deficient in selenium, confirm this pattern [61].

## Figures and Tables

**Table 1:** Food and water intake by mice injected i.p. with SeNPs (~5 μg/g) 5 h prior to exposure to ionizing radiation at a dose of 7 Gy.

Treatment	Days after Irradiation						
	0	1	3	5	10	15	30
	Food/Water Intake, g Food/Water Intake, % *						
0 Gy	4.9/7.4	5.0/7.5	5.0/7.4	4.9/7.5	4.9/7.4	5.0/7.4	5.1/7.5
	0/0	+2/+1	+2/0	0/+1	0/0	+2/0	+4/+1
	(10)	(10)	(10)	(10)	(10)	(10)	(10)
7 Gy	5.0/7.5	3.7/4.2	3.5/2.7	3.0/4.0	1.9/3.4	-	-
	0/0	−26/−44	−30/−64	−40/−47	−62/−55	-	-
	(10)	(10)	(10)	(9)	(1)	(0)	(0)
0 Gy + SeNPs	5.0/7.4	4.9/7.5	4.9/7.5	5.0/7.4	4.9/7.4	5.1/7.4	5.0/7.4
	0/0	−2/+1	−2/+1	0/0	−2/0	+2/0	+2/0
	(10)	(10)	(10)	(10)	(10)	(10)	(10)
7 Gy + SeNPs	4.9/7.5	4.5/6.3	4.0/5.5	4.0/6.1	4.5/6.8	4.9/7.1	4.8/7.6
	0/0	−8/−16	−18/−27	−18/−19	−8/−9	0/−5	−2/+1
	(10)	(10)	(10)	(10)	(8)	(6)	(5)

Data are means for n animals; n is given in parentheses. *—to the beginning of the experiment.

**Table 2:** Changes in the mRNA level of some “stress” genes in the red bone marrow cells of mice after exposure to ionizing radiation and administration of SeNPs (5 mg/kg). The effect of Sands administration on the change in the number of many copies of a number of genes was investigated after 24 h. The average values are presented (n = 5. SD ± 10–20%). ↑—an increase in the mRNA level relative to the control of 0 Gy by more than two times. ↓—a decrease in the level of m RNA relative to the control of 0 Gy by more than two times.

Genes	Relative Gene Expression			
	0 Gy		1.5 Gy	
	Control	Se NPs	Control	Se NPs
HO-1	8.5 × 10^−3^	7.2 × 10^−3^	1.4 × 10^−3^ (↓)	4.3 × 10^−3^
HSP90	3.2 × 10^−2^	2.6 × 10^−2^	1.2 × 10^−2^ (↓)	3.3 × 10^−2^
NFkb	1.9 × 10^−4^	2.9 × 10^−4^	2.0 × 10^−3^ (↑)	7.9 × 10^−4^ (↑)
NRF2	1.0 × 10^−2^	0.5 × 10^−2^ (↓)	0.3 × 10^−2^ (↓)	1.2 × 10^−2^
Catalase	3.0 × 10^−3^	3.3 × 10^−3^	1.5 × 10^−3^ (↓)	1.8 × 10^−3^
SOD2	1.8 × 10^−6^	2.5 × 10^−6^	1.2 × 10^−6^	3.7 × 10^−6^ (↑)
Prx6	9.2 × 10^−3^	8.5 × 10^−2^ (↑)	1.1 × 10^−1^ (↑)	2.8 × 10^−2^ (↑)
Xrcc4	6.8 × 10^−4^	8.8 × 10^−4^	1.8 × 10^−3^ (↑)	1.4 × 10^−3^ (↑)
Xrcc5	6.3 × 10^−3^	5.5 × 10^−3^	9.3 × 10^−3^	1.9 × 10^−2^ (↑)
TNF-α	2.5 × 10^−3^	3.1 × 10^−3^	2.4 × 10^−3^	1.9 × 10^−3^
AP-1	2.3 × 10^−2^	2.2 × 10^−2^	0.6 × 10^−2^ (↓)	2.5 × 10^−2^
Ki67	6.1 × 10^−3^	4.9 × 10^−3^	5.2 × 10^−3^	3.5 × 10^−3^
IL6	2.0 × 10^−2^	2.9 × 10^−2^	1.8 × 10^−2^	1.4 × 10^−2^

## Data Availability

Data available on request due to restrictions, e.g., privacy or ethical.
